# A Comparative Case Study Analysis: Applying the HIPE Framework to Combat Harmful Health Information and Drive COVID-19 Vaccine Adoption in Underserved Communities

**DOI:** 10.3390/vaccines11061107

**Published:** 2023-06-16

**Authors:** Linda Desens, Brandon Walling, Anna Fiedor, Vanessa Howard, Zue Lopez Diaz, Katherine Kim, Denise Scannell

**Affiliations:** The MITRE Corporation, 7525 Colshire Dr., McLean, VA 22102, USA

**Keywords:** misinformation, disinformation, COVID-19 vaccine, vaccine hesitancy, underserved populations, health equity

## Abstract

This descriptive, observational paper utilizes the comparative case study approach to analyze the application of the HIPE™ Framework to two health campaigns addressing vaccine hesitancy in underserved communities. Exposure to inaccurate/misleading health information impacts vaccination adoption, especially for individuals with low health/digital literacy. Underserved groups—like minority, racial/ethnic, or rural populations—typically have lower literacy and higher rates of vaccine hesitancy. Grounded in persuasion and behavioral change theory, the Health Information Persuasion Exploration (HIPE™) Framework was applied to the Black/Haitian community in Miami-Dade, Florida and the Migrant Agricultural Worker Community in Central Valley, California. The campaigns addressed each community’s unique characteristics via Detect, Analyze, Design, and Evaluate phases of the HIPE framework. Both campaigns achieved their respective vaccine uptake goals. For Miami-Dade, over 850 vaccinations were administered (the goal was 800 vaccinations), and vaccination rates increased by 25.22%. In Central Valley, vaccination rates for 5–11-year-old children in Merced and Stanislaus counties increased about 20% and 14%, respectively, and overall vaccination rates increased compared to surrounding counties. Discussion of the results and recommendations for future research highlight the potential efficacy of applying the HIPE™ Framework for developing health campaigns and response strategies to improve health outcomes.

## 1. Introduction

### 1.1. Inaccurate/Misleading Health Information and Its Impact on COVID-19 Vaccine Adoption

Vaccines are a safe and effective way to prevent serious illness. However, as of July 2022, only 67.7% of the United States population was vaccinated against COVID-19 [1]. Compared to other high-income countries like Australia (85.33% vaccinated), Canada (82.33% vaccinated), and the United Kingdom (74.25% vaccinated), this rate is relatively low. Vaccine hesitancy, or “delay in acceptance or refusal of vaccination despite availability of vaccination services”, contributes to the country’s low vaccination rate [2].

Recently, the World Health Organization announced that in addition to the COVID-19 pandemic, we are experiencing an “infodemic”, or overflow of harmful and misleading information [3,4]. Much of the health information circulating in the online environment is inaccurate and misleading. Particularly, mis/disinformation regarding the benefits and risks associated with COVID-19 vaccines has fueled hesitancy [5].

The literature shows that inaccurate/misleading health information can have serious effects on the issue of vaccination intention and adoption. A recent pre/post case control study found that those cases exposed to misinformation had a 6.4 percentage point decrease in intent to vaccinate against COVID-19 [6]. A separate study surveyed 600 Florida residents on their exposure to COVID-19 misinformation themes and their vaccination status/intentions [7]. The results showed that exposure to even one misinformation theme resulted in the individual being less likely to be vaccinated, finding that 73% of participants who did not report any exposure to misinformation were vaccinated, compared to 62.9% of participants that reported exposure to just one misinformation theme, and only 52.2% of participants that reported exposure to six or more themes, showing a significant correlation between vaccination status and exposure to misinformation themes. Of particular concern is the impact of inaccurate/misleading health information on individuals with lower health literacy, who are more susceptible and, thus, more likely to endorse misinformation and conspiracy theories [5,8]. One such group that is particularly vulnerable to influence by inaccurate/misleading health information are underserved populations like minority racial/ethnic groups and those living in rural areas [9].

### 1.2. Inaccurate/Misleading Health Information and Its Impact on Underserved Populations

Minority racial/ethnic groups are often exposed to more misinformation than their white counterparts [6]. In addition, these underserved populations often have less resources to combat it. In a study evaluating Promotoras’ (community health workers) perspectives regarding the COVID-19 vaccine in the Hispanic communities of Los Angeles, researchers uncovered various themes related to high prevalence of misinformation, vaccine hesitancy, and improvements needed for vaccination uptake [10]. The main barriers for vaccination against COVID-19 included a lack of trustworthy information, mistrust in the government, conspiracy theories, hesitancy based on health and safety concerns, eligibility confusion, accessibility issues, fears of cost, and immigration/deportation concerns. In Delaware, vaccination hesitancy and reduced COVID-19 vaccination uptake were major issues experienced by underserved communities due to lack of trust, vaccine misinformation with limited knowledge of the safety and effectiveness of the vaccine, how it works, and the overall push for getting the vaccine [11].

Various studies have demonstrated the differing levels, predictors, and causes of general vaccine hesitancy as well as hesitancy specific to COVID-19 across racial and ethnic groups. Reasons for general vaccine hesitancy among underserved populations include historical distrust in government agencies, pharmaceutical companies, doctors, and researchers that date back to the Tuskegee study, Nazi concentration camp studies, and measles/smallpox epidemics among Tribal Nations [12,13]. Tailored anti-vaccine campaigns aimed specifically toward the Black population and other minority groups have also contributed to general vaccine hesitancy [14]. Anti-vaccination organizations have used historical injustices and social history anecdotes as proof against vaccines. For example, anti-vaccination campaigns have attempted to link vaccination history to autism in order to increase vaccine hesitancy among minority populations [14]. These targeted campaigns further illustrate how effects of racism and other aspects of social determinants of health can impact the ways in which different groups within the general population interact with the landscape of health information to which they are exposed. Specifically for COVID-19, vaccine hesitancy among Black participants has been measured as high as 74%, and as high as 60% among Hispanic participants, compared to the national average of 26% [15,16]. Khubchandani et al. [16] reported on the major predictors of COVID-19 vaccine hesitancy for Black individuals and Hispanics and found that sociodemographic characteristics (e.g., younger age, female gender, lower income/education, and larger household sizes), medical mistrust, history of racial discrimination, and greater exposure to myths and misinformation are major contributors for these groups. For these historically underserved populations, vaccine safety has been reported as the top reason for COVID-19 vaccine hesitancy [9,15]. 

Moreover, underserved populations typically have lower levels of health literacy and digital health literacy [9,17]. Health literacy is the ability to understand and act upon health information [18], whereas digital health literacy refers to the ability to find and understand online health information to inform decisions [19]. In previous research, low digital health literacy has been linked to poor health outcomes [20]. Social determinants of health such as race, ethnicity, and area of residence contribute to differences in literacy, health inequities, and vaccine hesitancy. The literature suggests that Black and Hispanic populations with lower health literacy are more susceptible to misinformation [9]. Similarly, rural populations tend to have lower levels of health literacy as compared to urban and suburban populations [9,21,22]. Individuals living in rural communities are 10% less likely to get the COVID-19 vaccine [23]. Health literacy is complex, and there are multiple aspects of life that influence how individuals respond to health information. 

### 1.3. HIPE™ Framework 

Addressing inaccurate/misleading health information requires a systems approach to not only detect and identify inaccurate/misleading health information, but also to understand the audiences of interests and the array of variables that can impact health decisions and outcomes, as well as persuasion drivers or triggers used in the messaging. The Health Information Persuasion Exploration (HIPE™) Framework offers a systems approach to detection, analysis, message design and evaluation to address inaccurate/misleading health information with the ultimate goal of improving health literacy [24].

**Figure 1:**
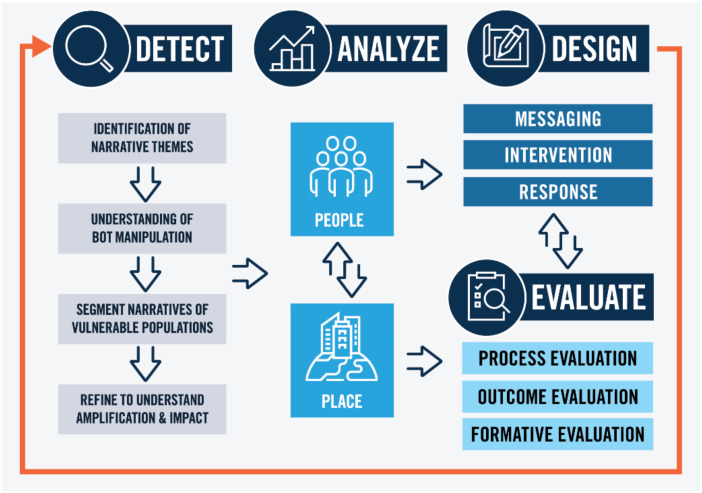
HIPE Framework Visual.

In 2020, a study was conducted to understand persuasion tactics used in COVID-19 vaccine messaging [24]. The study focused on persuasion tactics used in messaging in the three types of COVID-19 vaccine sentiments—Pro-Vaccine, Anti-Vaccine, and Neutral. Additionally, the study reviewed persuasion tactics used by inauthentic accounts, such as social media bots, used to artificially amplify online information and associated sentiment. From that research, the Health Information Persuasion Exploration (HIPE™) framework was developed to identify harmful health information and persuasion tactics or triggers used in COVID-19 anti-vaccine messages and provide a path forward in the development of rapid response counter strategies and interventions (Figure 1). 

The HIPE™ Framework establishes the critical role that persuasion patterns or triggers play in creating insightful, evidence-based strategies and responses to ensure accessible and equitable communication for underserved populations. The framework includes four key areas: detect, analyze, design, and evaluate. The detect phase includes the use of social media listening tools to identify narratives associated within health topic areas. Customized search parameters within these tools help to identify the narrative trends, amplification within these trends, as well as if these narratives are being artificially amplified. A key element within the detect phase is the identification of specific persuasion tactics or triggers being used to drive amplification which utilizes HIPE™’s persuasion algorithm [25]. The persuasion tactics or triggers are grounded in health communication theoretical frameworks—Extended Parallel Process Model, Social Judgment Theory, and Elaboration Likelihood Model—which include elements of information processing, social factors, as well as self-efficacy [26,27,28]. The analysis phase applies this intelligence within the attributes of people and place [29]. The attributes of people include individual factors that influence health such as demographics, language, and social norms, beliefs, and skills. “Place” includes attributes such as location of services, insurance, and transportation. This information is used in the next phase of the framework, the “Design” phase, to build evidence-based, precision response strategies and messaging that are customized to the audience of interest. The evaluation phase identifies if the recommended strategies are effective and highlights opportunities to refine the response strategy. Together, the elements of the framework create a holistic, data-driven approach to understand how individuals are exposed to and affected by harmful health information and how it varies across populations and geographies. Additionally, these tailored interventions and ongoing evaluation approaches enable a deeper understanding of specific population information needs and opportunities to identify a threat before it amplifies.

The purpose of this paper is to evaluate the utility of applying the HIPE™ Framework locally in the context of COVID-19 vaccine adoption in underserved communities and compare its application in two different communities -the Black/Haitian community in Miami-Dade, FL, USA and the Agricultural Worker community in Central Valley, CA, USA.

## 2. Methods

This descriptive, observational paper utilizes the comparative case study method to understand how the application of the HIPE™ Framework [24] to community-focused communication efforts could potentially impact vaccination rates [30]. The case study is a qualitative methodology used to explore a case or multiple cases within a time period using different sources of information and summarizing observations in a description of the case(s) [31]. Using this method, we will compare efforts that took place in two underserved communities: the Black/Haitian community in Miami-Dade County, Florida and the Migrant Agricultural Worker Community in Central Valley, California. The comparative case study approach allows for the comparison of multiple cases to better understand similarities and differences within given boundaries [32]. Using this approach, we observed and reported on the application of the HIPE™ Framework to community public health campaigns around COVID-19 vaccines in two different underserved communities in different areas of the country. 

The goals of the two campaigns were different—in Miami-Dade County, the goal was to administer 800 vaccinations (i.e., vaccine uptake) to members of the Black and Haitian Church community by the end of December 2021, whereas the goal in Central Valley was to increase vaccination rates in the agricultural worker community with a focus on the 5–11-year-old group in Stanislaus and Merced Counties. Additionally, the methods for applying each phase of the HIPE Framework were different, each campaign implemented the phases based on the unique characteristics of each population. In the following sections, an overview of the two campaigns will be presented.

### 2.1. The Communities

#### 2.1.1. Miami-Dade

In July 2021 through September 2021, MITRE began collaborations with Florida International University (FIU). The collaboration included Florida International University’s Keeping the Faith to Fight COVID-19 (KTFF) grassroots campaign, which officially began in May 2021, to promote vaccine adoption and reduce vaccine hesitancy through education and outreach. KTFF’s goal was to administer 800 vaccines to members of the Black church communities (including Haitians, English-speaking Caribbeans, and southern Black individuals) in Miami-Dade County by the end of December 2021.

KTFF primarily worked with the Black churches in the Miami-Dade area. The team conducted mobile vaccine outreach events in the local communities. They also offered regular education sessions on various health topics such as COVID-19 to local church leaders and one-on-one education at vaccine sites.

#### 2.1.2. Central Valley 

The second project was a collaboration with ACTIVATE [33] demonstration and research project for telehealth-enabled solutions to address vulnerable populations in rural and underserved communities impacted by COVID-19. The timeframe for this project was from February to June 2022. The goal was to apply the HIPE™ Framework to the rural and agricultural community in Stanislaus and Merced counties in Central Valley to increase vaccination rates among underserved communities, particularly in the 5- to 11-year-old group. This rural community consists of a preponderance of agricultural worker families, who are primarily Hispanic, with representation of Punjabi and Hmong community members. 

The team partnered with Livingston Community Health (LCH) and Valley Onward in support of their outreach activities to increase vaccine adoption. Each possesses a strong community outreach capability with the LCH resource specialists as well as the Valley Onward volunteer community health workers (CHWs) or Promotoras. This robust network of outreach workers offers the unique opportunity to build a trusting relationship with community members as they go from door to door to disseminate health information to community members.

## 3. Results

This section discusses the outcomes observed from the application of the HIPE™ Framework to the two campaigns focused on increasing COVID-19 vaccination adoption in Miami-Dade and Central Valley. The application of each phase of the HIPE™ Framework—Detect, Analyze, Design and Evaluate—are discussed for each of the aforementioned counties, with a comparison of similarities and differences between the applications.

### 3.1. Detect

Detection of online inaccurate/misleading health discourse around COVID-19 and the vaccine was collected and monitored using MITRE’s Social Integrity Platform™ using Talkwalker [34], a social listening tool and hosted ecosystem that provides inaccurate/misleading information threat detection. Although national detection of such discourse is readily available, it is a challenge to obtain this data at a local community level. Customized searches for online mentions of “vaccine” and its variations such as “booster”, “vax”, and “jab” were combined with filters on specific state counties within our communities of interest. These Boolean techniques and geo searches were used to capture the online discourse on COVID-19 and vaccines specific to those areas. The list of media types was extensive (e.g., Twitter, online news, blogs, newspaper, TV/Radio, Forums, Disqus, Magazine, YouTube and others). The majority of the data collected came from Twitter, as more content on the topic of vaccinations appeared on that platform than in news and blogs, and there is more publicly available data from Twitter than from private platforms such as Facebook. The results of the online discourse were categorized into the predominant themes related to the COVID-19 vaccine discourse. No individual-level or identifiable data was collected.

#### 3.1.1. Miami-Dade

Online social media data was collected for Miami-Dade from July 1–17 September 2021. 48,400 posts were collected across numerous online platforms. Most of the posts were from Twitter (57.7%), followed by news (20%), blogs (12.7%), and remaining posts came from newspapers, television, forums, Disqus, online Magazines, YouTube and others. 

To further support findings for the targeted online searches, “on the ground” strategies for crowdsourcing community discourse regarding COVID-19 vaccines further confirmed findings via online strategy and aided in the discovery of themes and issues that were missed. With the support of FIU, a mobile app that enables rapid social situational awareness of COVID-19 misinformation through crowd-sourced reporting, called SQUINT™, was implemented. Because the Social Integrity Platform™ is not able to access certain online platforms like WhatsApp, a popular communication vehicle for community members, it was necessary to find another option for capturing this discourse. In partnership with KTFF, SQUINTers were recruited from participating churches to help to detect and report inaccurate/misleading health information that they saw online. MITRE developed custom “SQUINTSTAGRAMS” to address inaccurate/misleading health information, submitted to SQUINT™ by participating church members and KTFF team members.

#### 3.1.2. Central Valley

For the Central Valley project, there were a total of 753,000 posts from February 2 to June 5, 2022. Like Miami-Dade, most of the posts were from Twitter (94.2%), followed by online news (3.8%), blogs (1.4%), newspaper (0.3%), and the remaining posts from other sources. The team opted to use WhatsApp for reporting inaccurate/misleading health information shared by personal communication from family and friends since SQUINT™ was not available for use at the time. The CHWs were selected to use the app to report inaccurate/misleading health information. Although past assessments identified WhatsApp as a popular platform for communicating among community members, the CHWs did not use it to report inaccurate/misleading health information, but instead shared insights on vaccine discourse from community members via one of our message testing sessions. Although crowdsourcing can be a valuable resource in obtaining information regarding COVID-19 vaccines, it is important to understand the best resources and tools for reporting within a designated community. 

### 3.2. Analyze 

#### 3.2.1. Analysis of Online Discourse 

An analysis of the online discourse was conducted to better understand the themes/narratives around COVID-19 vaccines at the local level so that the appropriate response strategies and messaging could be designed. The predominant narratives in Miami-Dade included: debate about vaccines for kids in schools (34.3%); vaccines will be forced/mandatory (27.8%); religious beliefs impact vaccine decisions (14.8%); new variants are emerging (14.3%); vaccines may have health consequences (8.8%). Posts related to religious beliefs included references to COVID-19 as a “plague for world leaders who made their communities or countries suffer”. Other posts urged people not to take the vaccine because it was “dirty” or would turn them into “vampires or zombies”. Conspiracy topics included discourse about high-tech government plots and population control.

For Central Valley, the main narratives found in the discourse included vaccine mandates infringing upon their personal freedom (31.8%), adverse effects of the vaccine (29%), conspiracy (23.2%), and vaccines are ineffective or not necessary (16%). Earlier during the reporting period, the narrative on the vaccine being ineffective and unnecessary was focused on the argument that kids should not be vaccinated. This narrative is reflected in the low vaccination rates for the 5-to-11-year-old group in Central Valley. With a goal of increasing vaccination rates in this age group, understanding the discourse around fears and values surrounding vaccinating children helped to inform the message design on this theme for parents.

Although data collection and observations for Miami-Dade and Central Valley took place during different times of the pandemic, both time periods shared narratives around the concern over loss of personal freedom through vaccine mandates and adverse effects of the vaccine. In Central Valley, the discourse on autonomy or personal freedom became a topic of focus e particularly during the national discussion of the overturning of Roe vs. Wade [35] Religious values and the connection to their local church community were reflected in the online discourse with Miami-Dade, but not Central Valley. Knowing this, partnering with local church pastors as the trusted messengers for delivering the message made sense for the Miami-Dade KTFF program. In contrast, this type of focus on religion was not seen in online discourse within Central Valley.

#### 3.2.2. Analysis of People and Place

In addition to the analysis of the online discourse, the research team conducted an analysis of people and place [29]. The analysis of people focuses on understanding the community members such as their social norms, values, health beliefs, fears, social networks, social determinants of health, language barriers, and health and digital literacy levels. Whereas the analysis of place focuses on the environment in which they live to include considerations such as the availability and accessibility of health services, transportation and insurance coverage. These factors are taken into consideration to better understand their impact on an individual’s health decisions.

##### Miami-Dade

When assessing the people and place aspect of a community, we also seek to better understand barriers and opportunities for health communication interventions. Barriers to people in the Black/Haitian community in Miami-Dade included low digital and general health literacy in the older population. Informal discussions with program outreach workers revealed that this generation preferred to listen to the radio instead of reading materials. 

Another barrier for this community included fears about the COVID-19 vaccine. Although the FDA granted emergency use authorization for COVID-19 vaccines, the community did not see this as the same full FDA approval as other vaccines and medications currently on the market. Fears related to discourse around the vaccine’s negative effects on fertility also circulated in the community. Finally, another major fear for this community, specifically for those who are undocumented, was the fear of repercussions (i.e., deportation) if they were vaccinated. Messaging needed to emphasize that the COVID-19 vaccination was open and free to all and did not require identification or insurance. 

Important to assessing the barriers to place or environment, there is limited or no broadband connection in the Little Haiti area. For the older Haitian population, this resulted in their use of radio as their primary source of information or obtaining online information from second-hand sources, such as family and friends. For the KTFF outreach team, another barrier was a minimum on the number of registered participants required to host a vaccine event within the county. Also, some areas did not allow the use of churches as vaccination sites. This required KTFF to seek other sites such as local parks that are accessible to multiple churches.

The assessment results showed opportunities for the best ways of communicating with the local community. The most used communication vehicles identified were WhatsApp and email. Pastors reported using email as the primary means of communicating important information to members of the congregation. They also used the church bulletin or used announcements on their big screen at the church to disseminate information just prior to the service. Because of low bandwidth and the preference of the older population to listen to the local radio stations for information, public service announcements were identified as a communication vehicle for this audience. WhatsApp was identified as a primary communication vehicle that community members used to communicate with each other. A final consideration related to communicating with the audiences of interest is the preferred languages. For this community, the preferred languages were English and Haitian Creole. This understanding informed the importance of developing communication assets in both these languages.

One very important factor in the analysis of people and place was understanding who community members trusted for health information. Trusted messengers included the pastors as well as the local healthcare providers. KTFF offered regular education sessions via webinar and in person at COVID-19 vaccine events leveraging recognized local health care providers in the community. By attending the vaccine events in person, the providers were able to engage with community members one-on-one to answer any questions and address any concerns about the vaccine. Leveraging both of these influential messenger groups in the community enabled KTFF to use these trusted relationships to share information about the COVID-19 vaccine to decrease vaccine hesitation and increase vaccine adoption.

##### Central Valley

Like the target audience in Miami-Dade County, community members of Stanislaus and Merced counties were concerned about possible adverse effects of the COVID-19 vaccine. Rather than generational fear and trauma like in Miami-Dade, these community members were more concerned about becoming sick from the vaccine and not being able to work and support their families, which is highly valued by this community. Low COVID-19 vaccine rates for 5–11-year-olds in this community were linked to doubts about the safety of the vaccine.

The top sources for obtaining health information for this community included social media, word of mouth, their children’s school, and their doctor. LCH and Valley Onward sent CHWs into the local community to share information. Outreach workers canvassed specific zip codes within the county going door-to-door talking to residents about health-related issues including information about COVID-19 and the vaccines. Residents welcomed them into their homes providing opportunities for creating trusted relationships with the outreach workers. Because the Promotoras were volunteers from the community, they had a special connection with the Hispanic agricultural workers. They understood the social values and norms, the culture, the language, and the people. They were the trusted messengers, who community members welcomed into their homes. Other populations in these counties included Punjabi and Hmong. Addressing the needs of these cultures was also a priority for outreach workers.

In the assessment of “place”, public health workers related challenges with communicating with the employers. Employers were not as open to hosting vaccine distribution at the worksite. However, there was a strong partnership with the schools. This allowed for easy access to vaccination distribution sites at the schools. For areas with low vaccine rates, mobile vans were deployed to facilitate access to the vaccines.

The analysis of online discourse and assessment of people and place provided valuable insight that informed the design and development of the response strategies and the design of the communication assets.

### 3.3. Design

The health communication team focused the design of communication assets and response strategies based on the findings and themes identified in the Detect and Analyze phases. Results of the analysis of people and place also helped to better understand the best communication vehicles, influencers, or messengers to reach the audience of interest with health information on the COVID-19 vaccines. The persuasion algorithm that was developed as a result of the HIPE™ Framework provided insights as to the persuasion drivers used in the online discourse that are being implemented to influence health decisions [24]. These included persuasion drivers such as social values, information processing (i.e., peripheral or central processing) and fear appeals, response efficacy, and self-efficacy.

For both Miami-Dade and Central Valley, the team took into consideration the different audiences of interests in the communities. For example, KTFF wanted to not only reach the higher risk population such as the elderly, but also young men in their 20s, who had very low vaccination rates when compared to other age groups in the county. Communication assets were customized with messaging specific to each subpopulation as well as the preferred languages.

There was a total of 121 communication assets created in English and Haitian Creole for Miami-Dade. These included communication assets for social media, WhatsApp, email and church bulletins and announcements, as well as public service announcements. For Central Valley, with a goal to increase vaccination rates in 5–11-year-olds, messages were designed to reach parents through the local schools and social media. A total of 65 communication assets were developed that included social media graphics, email/text blasts, flyers, and post cards in English, Spanish, and Punjabi. The program coordinators expressed the value in being able to leave postcards and flyers behind during the community canvassing. They stated that it gave them a starting point for a conversation and something to leave behind at the end of the visit. A summary of communication artifacts can be found in Table 1.

### 3.4. Evaluate

#### 3.4.1. Miami-Dade

Although the HIPE™ framework consists of four types of evaluation—process, outcome, formative, and impact—this case study focuses on the formative and impact evaluation results. In Miami-Dade, county vaccination rates at the beginning of the observation in July 2021 showed 56.3% of the population fully vaccinated and by the end of observation period in September 2021, showed an increase of 25.22% positive percent change and a fully vaccinated rate of 70.5%.

For Miami-Dade, efforts consisted of a collaboration with Keeping the Faith to Fight (KTFF), a community-based, grass roots effort. The overall objective for this campaign was to promote vaccine adoption and to reduce vaccine hesitancy through education and outreach. The primary campaign activities consisted of mobile vaccine outreach events and regular education sessions to connect with pastors and community health workers. These activities included developing and distributing reports on themes found in the online discourse related to COVID-19 vaccines; developing messages in English and Haitian Creole via communication vehicles such as Facebook, Instagram, WhatsApp, emails, bulletins, PSAs, and social media graphics; conducting formative evaluation or informal listening sessions with community health care workers and pastor groups to test messaging designs and communication artifacts. The campaign collaboration with KTFF also involved using SQUINT™, which enabled individuals in the local communities to report inaccurate/misleading health information that they experienced in the church community to develop community specific SQUINTSTAGRAMs to share relevant and accurate COVID-19 vaccine information through WhatsApp and other social media platforms. 

Findings from formative evaluation sessions with pastors from 28 Majority-Black churches and community health care workers in Miami-Dade were used to inform the revisions of messaging to ensure they were relevant to the local Black and Haitian community. Results of this message testing focused primarily on tone, content, values, language, and delivery method. Churches in this community primarily reported using the telephone and email as primary means of communication, and the older generations listen to radio rather than read content on social media or other mediums. The social media app WhatsApp is another popular communication vehicle that the Haitian church community reported using for sharing information and internet videos. Participants in formative evaluation sessions recommended using pastors and other community leaders as key messengers. Additional recommendations included simplifying messages, emphasizing the importance of being educated on the facts to enable individuals to make their own decisions about getting vaccinated, and focusing less on ‘getting back to normal’ and more on the protective factor of getting vaccinated. 

The KFF campaign exceeded its goal of administering over 850 vaccines via KTFF events before the end of the year. In addition to the goal of distributing vaccines, an overall objective was to promote vaccine adoption and reduce vaccine hesitancy in Miami-Dade. Considering fully vaccinated rates for adults over 18, in September 2021 Miami-Dade had passed 80%, which is around 10–15% higher than neighboring counties (Broward, Monroe, and Collier) [36]. 

#### 3.4.2. Central Valley

The application of the HIPE™ framework to outreach efforts in Central Valley, California was a collaborative effort to support LCH and Valley Onward, which service zip codes within Merced and Stanislaus Counties. The application of the HIPE™ framework in Central Valley, California was a collaborative effort to support LCH and Valley Onward, which service zip codes within Merced and Stanislaus Counties in Central Valley. There was a total of three message testing sessions that were conducted over the Zoom platform—two with LCH and one with the Promotoras. The session with the Promotoras was led by a team member who was fluent in Spanish and had a strong knowledge of the project. She was able to facilitate a robust discussion with the group where the participants openly shared their feedback of what they learned about the community and their attitudes about the COVID-19 vaccine from their home visits. The discussions were key in understanding the right messaging efforts for the community and resulted in insights as to how to best reach them. A limitation in the formative evaluation was the lack of opportunity to test the messaging with the Hmong and the Punjabi communities due to lack of access.

To analyze the impact COVID-19 vaccine outreach efforts had on their residents, we assessed the change in vaccination rates using publicly available data from the beginning to the end of the observation period and compared areas where the outreach efforts were conducted to areas not receiving outreach efforts. LCH provided the zip codes for the intervention areas. Using data collected by the California Department of Public Health and accessed through the California Health and Human Services Open Data Portal [37], we were able to explore changes in vaccination rates by zip code, county, age group, and further by difference in vaccination status. The date range for this data ran from 14 March to 30 June 2022, for county-wide data and 15 March to 28 June 2022, for zip code-specific data. Vaccination status was described as either 1 dose + (having 1 or more doses), fully vaccinated (two doses of Pfizer or Moderna), and booster eligible (fully vaccinated plus at least 1 booster). 

For county-wide all population full vaccination rates, Merced County experienced the highest increase compared to other Central Valley counties where interventions did not take place (percent increase = 2.48%). Both non-intervention counties and intervention counties experienced statistically significant increases in all county vaccination rates from start to end of the intervention (Table 2). Despite not reaching statistical significance, intervention counties (average percent increase = 2.25%) experienced higher increases from start to end of the intervention compared to non-intervention counties (average percent increase = 1.84%).

Based on the CDC’s [38] November 2021 recommendation that 5–11-year-old children (approved for Pfizer) be vaccinated, the main goal of the intervention in Central Valley was to positively influence and support community engagement with the action of getting children vaccinated. In Merced County, the rate of full vaccination for this age group increased from 13.63% to 16.29% which had a positive percent change of about 20% (19.57%) from March to June 2022. In Stanislaus County, the full vaccination rate for 5–11-year-old children increased from 14.21% to 16.23%, resulting in a positive percent change of 14.22% from March to June of the intervention period. Compared to other counties around Central Valley where the intervention did not take place (in Butte, Fresno, Tehama, and Tulare counties), non-intervention counties had lower percent changes from beginning to end of the intervention period ranging from about 12% to 13% positive percent change. Both non-intervention counties and intervention counties experienced increases in 5–11-year-old vaccination rates, however the change in the intervention group did not reach statistical significance (see Table 3). Despite not reaching statistical significance, the impact on this 5–11-year-old age group in regard to full vaccination status was evident based on substantively higher increases in Merced and Stanislaus counties where interventions took place (average percent increase in Merced/Stanislaus = 16.81%) compared to non-intervention counties (average percent increase = 12.54%)

In analyzing COVID-19 vaccination data based on zip codes, we found that residents serviced within the intervention zip codes had steady increases of growth hovering at about 2% to 3% positive change, while the zip codes outside of the intervention had larger gaps between them. All zip codes experienced a statistically significant increase in vaccination, with Merced (zip code: 95341) experiencing the highest percent change increase of 2.92% (46.69% to 48.06% fully vaccinated). Evidence of the impact of LCH and Valley Onward interventions, zip codes where interventions took place saw the greatest increase compared to zip codes in Merced County where interventions did not take place and zip codes in Stanislaus County where interventions did not take place (Table 4). Further, the difference between fully vaccinated rates by zip codes where LCH and Valley Onward interventions took place was significantly higher compared to non-intervention zip codes in Merced County and non-intervention zip codes in Stanislaus County. Consistent growth among zip codes where Livingston Community Health held vaccination events and Valley Onward canvassed the community shows a positive trend of reaching its residents and encouraging vaccination throughout all the zip codes they reached.

## 4. Discussion/Implications for Practice

The current comparative case study analysis describes the application of the HIPE™ Framework for detecting inaccurate/misleading health information and informing response strategies and messaging at the local community level. Grounded in persuasion and behavioral change theory, persuasion drivers were analyzed at the local level by observing and understanding the kinds of inaccurate/misleading health information that the specific communities are exposed to. Communication assets were designed with the objective of addressing the inaccurate health information themes uncovered in the local discourse and also tailored for the specific population needs. Finally, evaluation of intervention efforts included: formative evaluation via message testing and listening sessions with relevant groups of community stakeholders; process evaluation with community partners; outcome evaluation in regard to administering vaccinations via community engagement events; and impact evaluation, assessed by comparing vaccination rates where interventions took place to vaccination rates in surrounding areas where interventions did not take place. In addition to validating the HIPE™ Framework and translating research about inaccurate/misleading health information into practice by disseminating theoretically-based messages to relevant subgroups of the population, findings of this comparative case study analysis have important implications for public health campaigns—notably, that precise communication assets need to be developed for the specific target audiences where effects are desired, the necessity of evaluation and understanding the communities that campaigns intend to reach, and for equity and communication.

### 4.1. Precision Communication

Previous research has demonstrated the effectiveness of targeted and tailored public health campaign messages compared to generic “one size fits all”, homogenous messages when it comes to reaching target audiences [39,40]. Often, only a subgroup of the total population needs to be reached by any specific message [41]. When it comes to strategic message design, the degree to which messages are customized to their target audience can vary—from non-customized, mass-produced messages to more strategically designed messages that use audience segmentation principles to develop theoretically-based messages using varying levels of customization [27,40,42]. Health campaigns can optimize their effectiveness to combat misinformation by adapting message variations to match important aspects of their target audiences—such as their health literacy or their attitudes about relevant health issues—following the National Institute of Health’s “Precision Medicine Initiative” and expanding the concept to include “precision communication” [43].

For both campaigns included in the current comparative case study analysis, communication assets were designed based on the detect and analyze steps of the HIPE™ Framework. Analysis of the people and place was conducted to understand the people in the community and their social norms, values, and fears related to COVID-19 and vaccination, how those attitudes interact with their physical environment, and how these factors impact their behavioral decisions, which gave the opportunity for developing tailored messages for the different audiences. An additional consideration were the persuasion drivers identified in the online discourse. In Miami-Dade, messages were developed for parents, for young men in their 20s who were showing low vaccination rates, and for individuals with concerns about fertility. In Central Valley, the goal was to increase vaccination rates in the 5–11-year-old age group; therefore, messages were created to target parents. These strategies are in line with the literature on health communication campaigns, such as the “Let’s Move” campaign, for example, which applied the Extended Parallel Process Model [44] to persuade parents with danger control messages that indicate high levels of perceived threat and high levels of efficacy to influence their children’s behavior [45].

By identifying different subgroups within a population, information about shared determinants of behavior can be utilized to craft persuasive messages for different subgroups based on factors such as perceptions of risk for health issues [27]. (For example, an effective message targeting individuals that perceive a high risk for COVID-19 might not be as effective among individuals that perceive a low risk for COVID-19. By doing work to understand how the target subgroups think and feel about the relevant health issue, strategic messaging should be used to enable some level of customization to fit the audience based on some psychological, social, cultural, or behavioral similarity [40]. Culturally relevant and specific messages are rated as more credible, attractive, and higher quality than generic messages—by increasing the cultural appropriateness of messages, those messages will resonate more with the target audience and increase the chance of having the desired effect.

### 4.2. Necessity of Evaluation

An important element of any communication campaign is to conduct evaluation to determine the effectiveness and impact of any intervention effort [46]. The criteria to determine effectiveness is not standard for all interventions, rather, it is important to define what constitutes success for all campaign efforts early on. Some efforts might require robust measurement of specific variables before and after any intervention event or over long periods of time to determine effectiveness, yet others might have immediate impact that can be attributed to campaign effectiveness. Improvements in health indicators that are consistent with the messages from a health communication campaign are one sign that a campaign had an impact, along with population-wide changes in knowledge, awareness, or behavior advocated by the campaign [47]. Crucial to assessing impact, however, is understanding the objective of the efforts in regard to the target population. By using theory-based evaluation research, incorporating audience targeting and message design, health communication campaigns can determine which message strategies work for specific subgroups of the population [41]. Understanding the unique needs of specific subgroups within the community that messages are intended to reach is one way the field of health communication can work to address and reduce disparities among underserved populations with a goal of achieving health equity [48], which should be the primary concern that guides evaluation.

For each of the campaigns presented in this comparative case study analysis, the target population was different, and therefore, each had different objectives. For both, however, collaboration with community partners and the analysis of people and place enabled campaign efforts to understand the relevant attitudes of the target communities. One of the highlights of these case studies worth noting was the power of CHWs directly interacting with community members. This offered CHWs the opportunity to build trusting relationships. For example, the Promotoras in Central Valley went door-to-door in certain zip codes, reaching out to individual households to better understand their concerns about the COVID-19 vaccine, to share factual information regarding the vaccine, and to empower community members to be advocate for their own health.

Lessons learned from the current study offer practical implications for public health communication campaigns, especially concerning the importance of evaluation. First, it is important to conduct formative research to understand the nuances and needs of the specific communities that campaign efforts intend to reach [46]. Next, process evaluation enables campaigns to determine effectiveness as intervention efforts develop, opening up the opportunity to adapt strategies to better fit the target audience if the effects are not working out as planned. Outcome evaluation requires a deep understanding of the relevant community to set appropriate goals early on that matter for and fit the needs of the specific population then later, determining whether or not objectives have been met. Finally, impact evaluation builds on formative and summative efforts related to the campaign to assess the effects that interventions have in regard to change in awareness, attitude, or behavior among the target group as a result of the communication intervention [46,47].

## 5. Limitations

It is important to note that there are several limitations of the current study. First, because this was a descriptive, observational report, we cannot attribute the changes in vaccine rates strictly to the application of the HIPE™ framework. In addition to the described public health campaigns in the observed counties, individuals in each area were also exposed to countless other messages related to COVID-19 and vaccines in their unique information ecosystems—any of these messages could have also had an impact on individuals to either want to get vaccinated or not. Another limitation is that the observed results are not generalizable outside of the two respective populations with which the campaigns took place. For example, unique factors about each campaign and population, such as the community health outreach workers in Central Valley, who went door-to-door to community members’ homes and interacted with them directly, could be a primary reason for the success in vaccine rates in those specific counties. Also, community engagement by local healthcare providers and opinion leaders like the pastors in Miami-Dade County could have also had an impact because of the unique characteristics of the specific people and place. Another limitation in the design of the current study was the use of publicly available, open-sourced data to collect and observe changes in vaccine rates. Future research could include an experimental design that uses intervention and control groups to test for experimental effects of the HIPE framework/approach, or specific messages on different groups.

## 6. Conclusions

HIPE™ is a novel systems approach that uses both national and local online social media data to pinpoint inaccurate/misleading health information and provides insights to persuasion drivers in the online discourse to inform tailored communication response strategies and messaging. The comparative case study of the application of HIPE™ in two distinct communities offers a foundation for understanding the framework features that could potentially contribute to improved communication and health outcomes. Further, the application of HIPE™ in such different communities representing East and West coast, urban and rural geography, as well as varied racial/ethnic demographics and languages suggests the framework is promising as a potentially generalizable model. Future research and demonstration in other underserved communities will enhance the evidence to assess whether HIPE™ can fulfill this promise in the practice of precision, equitable health communications.

## Figures and Tables

**Table 1:** Communication Artifacts.

Audience of Interest	# Assets	Communication Method	Language	Examples
Central Valley, CA, USA (Stanislaus & Merced Counties)	65	Social media graphics Email/text blasts Flyers Postcards	English Spanish Punjabi	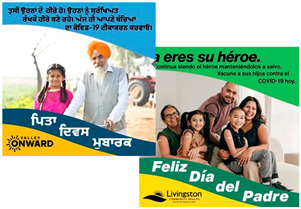
Miami-Dade County, FL, USA	121	Social media graphics WhatsApp messages Email blasts Church Bulletin posts Public Service Announcements	English Haitian Creole	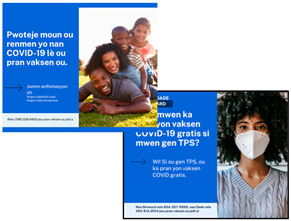

# represents number.

**Table 2:** County-wide all population vaccination rates: Merced/Stanislaus vs. surrounding counties.

		Start	End	*t* *	*p*
Merced/Stanislaus Counties		*(M* = 56.64, *SD* = 3.83)	(*M* = 57.92, *SD* = 3.75)	19.6	0.016
	Merced	53.93	55.27		
	Stanislaus	59.35	60.56		
Surrounding Counties		(*M* = 55.07, *SD* = 7.24)	*(M* = 56.08, *SD* = 7.47)	6.8	0.003
	Butte	54.04	54.79		
	Tehama	46.21	46.97		
	Tulare	56.2	57.47		
	Fresno	63.81	65.08		

Note. * Paired sample *t*-tests; independent samples *t*-test: *t* = 1.15, *p* = 0.155.

**Table 3:** 5–11-year-old vaccination rates: Merced/Stanislaus counties vs. surrounding counties.

		Start	End	*t* *	*p*
Merced/Stanislaus Counties		(*M* = 13.92, *SD* = 0.41)	(*M* = 16.26, *SD* = 0.04)	7.35	0.086
	Merced	13.63	16.29		
	Stanislaus	14.21	16.23		
Surrounding Counties		(*M* = 15.67, *SD* = 5.14)	(*M* = 17.63, *SD* = 5.83)	5.63	0.011
	Butte	15.92	17.83		
	Tehama	9.16	10.23		
	Tulare	15.87	17.98		
	Fresno	21.74	24.51		

Note. * Paired samples *t*-tests; independent samples *t*-test: *t* = 0.67, *p* = 0.27.

**Table 4:** All-population fully vaccinated rates: campaign zip codes vs. non-campaign zip codes.

		Start		*t* *	*p*
Campaign Zip Codes		(*M* = 53.97, *SD* = 5.84)	(*M* = 55.23, *SD* = 5.85)	27.74	<0.001
	Atwater	55.59	56.74		
	Delhi	51.18	52.29		
	Livingston	63.44	64.82		
	Merced	46.69	48.06		
	Modesto	56.36	57.6		
	Winton	50.58	51.84		
Non-Campaign Zip Codes - Merced		(*M* = 40.17, *SD* = 2.17)	(*M* = 40.94, *SD* = 2.5)	4.48	0.01
	Ballico	37.39	37.68		
	Hilmar	39.73	40.64		
	Snelling	41.05	41.86		
	Stevinson	42.51	43.61		
Non-Campaign Zip Codes - Stanislaus		(*M* = 47.51, *SD* = 4.2)	(*M* = 48.46, *SD* = 4.46)	4.85	0.02
	Hickman	42.66	43.32		
	Vernalis	50	51.32		
	Waterford	49.87	50.73		

Note. * Paired samples *t*-tests; independent samples *t*-tests: Merced *t* = 3.14, *p* = 0.014; Stanislaus *t* = 2.12, *p* = 0.036.

## Data Availability

Open-source data was collected from CHHS. Statewide COVID-19 Vaccines Administered by County 2022. Available from: https://data.chhs.ca.gov/dataset/vaccine-progress-dashboard/resource/130d7ba2-b6eb-438d-a412-741bde207e1c (accessed on 5 October 2022).
